# Masking Abilities of Dental Cad/Cam Resin Composite Materials Related to Substrate and Luting Material

**DOI:** 10.3390/polym14030364

**Published:** 2022-01-18

**Authors:** Liliana Porojan, Roxana Diana Vasiliu, Sorin Daniel Porojan

**Affiliations:** 1 Department of Dental Prostheses Technology (Dental Technology), Center for Advanced Technologies in Dental Prosthodontics, “Victor Babeș” University of Medicine and Pharmacy Timișoara, Eftimie Murgu Sq. no. 2, 300041 Timișoara, Romania; sliliana@umft.ro; 2 Department of Oral Rehabilitation (Dental Technology), Center for Advanced Technologies in Dental Prosthodontics, “Victor Babeș” University of Medicine and Pharmacy Timișoara, Eftimie Murgu Sq. no. 2, 300041 Timișoara, Romania; porojan.sorin@umft.ro

**Keywords:** resin-composite, masking ability, luting material, substrate

## Abstract

An outstanding treatment challenge related to aesthetic monolithic materials is to mask discolored substrates in aesthetic areas. The purpose of the study is to evaluate the substrate masking ability of different resin composite materials and the influence of their association with luting agents and substrates. Five types of 2M2 HT (high translucency) resin composite materials were selected: Vita Enamic [E] and four types of nanoparticle-filled composites Lava Ultimate [L], Cerasmart [C], Shofu HC [S], and Hyramic [H]. Resin composite Vita VM LC with different shades was used for the substrates: 2M2, 3M2, and CP2. Variolink Esthetic Try-inpastes neutral, light+, and warm+ colors were chosen to simulate the luting agent color. Optical parameters (TP (translucency), CR (contrast ratio), and OP (opalesce)) and color differences ΔE (chromatic difference) were calculated. Statistical analyses were performed to evaluate the comparisons between the groups and establish correlations. TP average values for all materials were in the range of 21.49–24.53. OP average values were in the rage of 6.31–7.85. OP is moderate positive correlated to TP and CR is negative and strong correlated to TP. Related to materials, average color changes decrease as following: E > H > C > L > S. Referring to the tryin material, warm colors induce marked color changes of the restoration. The differences of the color changes determined by all studied substrates are significant. For the final aesthetic aspect of the restoration, it is essential to consider the underlying dental structure, luting agent, and restoration material as a whole unit. The masking ability of the investigated resin matrix ceramic materials materials shows differences, the best behavior demonstrated Shofu HC and Lava Ultimate. Marked color changes are related to high chroma substrates. For substrates with a darker color, the association with warm try-in pastes lead to marked color changes, but with neutral and light try-in pastes at most perceivable.

## 1. Introduction

Due to the fact that patients’ requirements for aesthetic dental restorations are increasing, different material options have to be considered. Taking into account the fact that the trends towards monolithic restorations are more and more obvious, several types of materials have been developed for these purposes. Besides established ceramics and composite, the new generation of materials, resin matrix ceramic materials materials, a compound of the previous, was introduced as an alternative [1].

The physical properties of these materials are very close to those of natural teeth. Resin matrix ceramic materials materials are less brittle, less rigid, than ceramics, however are easy to repair. The fracture toughness, elastic modulus, hardness lie between ceramics and composites. They cause less abrasion of natural teeth than ceramic materials and the wear is lower than those of composites [2,3,4,5,6,7]. Due to these properties, these materials are increasingly required in practice. Resin composite materials are adequate to use in the anterior and posterior areas with the possibility of minimum structure resistance. In order to adjust the optical aspect, these resin composite blocks are available in three different translucency levels [8].

An outstanding treatment challenge related to aesthetic monolithic materials is to mask discolored substrates in aesthetic areas. The luting material color, the substrate color, and restoration material optical properties might have a significant influence on the final optical appearance of the restoration. Bilayered restorations, due to the high opacity of zirconia or lithium disilicate copings used for frameworks, should be veneered and the masking effect is no longer relevant. The use of different aesthetic materials for monolithic restorations is more common in order to avoid problems related to veneer chipping of bilayered structures and to decrease the technological stages and working time [9,10,11].

Alternative materials have been therefore developed, like high-translucent zirconia, reinforced silicate ceramics, or resin composites. From all of these materials, the last ones are preferred even in terms of processing, because they do no longer require firing for sintering or crystallization. Besides the practical use of monolithic restorations, their masking ability of discolored substrates is not elucidated in literature [11,12].

When evaluating the final aspect of aesthetic restorations, it is essential considering that the restoration will function as one body (underlying dental structure, luting agent, and restoration material). Taking this into account, a comprehensive determination of restoration material, luting agent, and background color together will optimize the color selection [13].

The color of the substrate cannot change, it can only be masked. The masking ability has been defined as the ability to hide a colored background. Masking a dental substrate with cements, after some authors, may not be feasible because different shades do not exist, and the cement layers are thin [14,15].

Related to the luting material, other studies consider it an important factor in aesthetic rehabilitation, and demonstrated that it affects approximately 10–15% of the optical results of all-ceramic restorations, and to obtain better aesthetic predictability, try-in pastes should be tested prior to cementation [16,17,18].

Taking these into account, the masking ability of the restoration material is very important. Studies proved that the masking effect can be correlated with the translucency parameters (TP) values when the TP values of the materials are different. However, when the TP values were in a similar range, the opalescence parameters (OP) may influence the masking effect. Therefore, the contribution of the opalescence should be confirmed quantitatively. Opalescence is caused by the scattering of the visible spectrum of light, giving the material a bluish appearance in reflected color, and an orange/brown appearance in transmitted color, because the shorter wavelengths of light are scattered more than the longer wavelengths [18,19,20].

Due to relative translucency of aesthetic materials, a substrate may compromise the masking ability of the restorations. Some studies have evaluated the optical behavior of different glass and zirconia ceramics and assessed besides the effect of various factors on the final color of the restorations including dental substrates, luting agents, restoration material, and glaze, and laboratory procedures [21,22,23,24,25,26,27,28,29,30,31,32,33]. Little information is available on the complex optical behavior of aesthetic CAD-CAM materials, related to the color parameters of natural teeth structures. Different optical properties increase the complexity of the color matching process because monolithic ceramic restorations allow more light to enter with increased scattering [34,35,36,37]. Different studies reported high translucency parameters of reinforced glass-ceramics, lower for polymer infiltrated ceramics, and while resin-based composites were evaluated to be also more translucent [38,39,40]. The most studies investigated glass ceramics, high translucent zirconia and composites. There is a lack of knowledge related to resin composites materials materials.

The purpose of the study consists of a comprehensive approach, in order to evaluate the substrate masking ability of different resin matrix ceramic materials materials and assess the influence of their association with luting agents and substrates. The first null hypothesis was that “the masking ability of resin composites materials materials shows no significant differences”, the second refers to the luting agent, namely “the luting agent affects the color of the final restoration”, and the third is related to the substrate, “the color of the substrate influences its masking ability”.

## 2. Materials and Methods

### 2.1. Specimen Preparation

Resin compsoites materials materials blocks were sliced in rectangular-shaped plates with 1 mm thickness (*n* = 8 for each group) using a machine (Orthoflex PI Dental, Budapest, Hungary). Five types of 2M2 HT resin composites materials were selected: Vita Enamic (VITA Zahnfabrik, Bad Säckingen, Germany) [E] and four types of nanoparticle-filled resins (Lava Ultimate, 3M ESPE, St. Paul, MN, USA) [L], (Cerasmart, GC Corporation, Tokyo, Japan) [C], (Shofu HC, Shofu, Kyoto, Japan) [S], (Hyramic Upcera, Liaoning, China) [H] (Table 1).

Specimens were polished using silicon carbide papers (600–2000 grit) and the final thickness of each specimen was checked with a digital caliper. They were finally polished [p] with a low-speed handpiece and diamond polishing paste Renfert polish all-in-one (Renfert, Hilzingen, Germany), cleaned with 98% ethylic alcohol, dried and divided in two halfes. Half of the specimens received no further surface treatment and the other half were glazed [g]. The purpose fotr this was to evaluate in which degree the glaze influences the optical properties of the samples compared to the polished samples.

Resin Glaze Primer (Shofu, Kyoto, Japan) was applied to the ceramic surfaces for 60 s, allowed to dry, and then two thin layers of glaze Resin Glaze Liquid (Shofu, Kyoto, Japan) were applied with a soft brush, in one direction to eliminate air bubbles and were polymerized for each 180 s in a light-polymerizing device Sibari Sr 620 (Sirio Dental, Meldola, Italy).

The substrates adopted in the study were rectangular shaped plates with 3 mm thickness. Composite resin Vita VM LC (Vita Zahnfabrik, Bad Säckingen, Germany) with different shades was used for the substrates: 2M2 (control), 3M2 (a darker color), and CP2 (a higher chroma). The color substrate was chosen to simulate as much as possible the tooth color.The material was applied in layers and light activated for 60 s.

In order to simulate the luting material, analyses were asessed with water-soluble glycerin pastes Variolink Esthetic try-in (Ivoclar Vivadent, Schaan, Liechtenstein), placed between the resin composites materials specimen and the background. Neutral (control), light+, and warm+ colors were chosen for the analyses. These analyses evaluated the potential of masking ability caused by the restorative material and a simulated luting agent. The try-in was applied between the resin composites materials specimen and the substrate with standardized thicknesses, by application of a 500 g load for 30 s.

### 2.2. Optical Investigations

Color parameters were obtained in three coordinate dimensions of L* (from 0 [black] to 100 [white]), a* green–red (−a* = green; +a* = red), and b* blue–yellow (−b* = blue; +b* = yellow).

The analysis of the color was done under a D65 illuminant using a spectrophotometer Vita Easyshade Advance 4.0 (Vita Zahnfabrik, Bad Säckingen, Germany). All measurements were carried out by the same operator, and under the same illumination, to exclude any variation. The Easyshade was recalibrated after each specimen.

The translucency parameter (TP) of materials was calculated with the Equation (1).

TP = [(L_b_ − L_w_)^2^ + (a_b_ − a_w_)^2^ + (b_b_ − b_w_)^2^]^1/2^ (1)

Related to this parameter, the lower the TP value, the more opaque the material is, providing higher masking ability [40,41,42].

OP values were calculated using the Equation (2).

OP = [(a_b_ − a_w_)^2^ + (b_b_ − b_w_)^2^]^1/2^ (2)

CR was achieved by Equation (3)

CR = Y_b_/Y_w_ Y = [(L* + 16)/116]^3^ × 100 (3)

w and b are color coordinates of the specimens on the white and black backgrounds. In this calculation, CR = 0 is considered transparent, and CR = 1 is regarded as totally opaque [40].

The Equation (2) was used to measure the color difference ΔE of each specimen on a discolored background in relation to the background 2M2 (control).

ΔΕ* = [(ΔL*)^2^ + (Δa*)^2^ + (Δb*)^2^]^1/2^ (4)

The national bureau of standards (NBS) system was used to quantify the levels of color change (Table 2). To relate the color change to a clinical standard, the ΔE* values were converted into NBS units: NBS = ΔE* × 0.92 [43,44,45,46].

### 2.3. Statistical Analysis

Statistical analyses were performed by means of the IBM SPSS Statistics software (IBM, Armonk, NY, USA). Average values and standard deviations (SD) were calculated. Paired *t* test was used to evaluate the comparisons between the means. A *p* value of under 0.05 was considered statistically significant. Spearman correlation was used to assess relationships between variables. It measures the strength of association between variables and the direction of the relationship (positive or negative). The significance was related to: 00–0.19 “very weak”, 0.20–0.39 “weak”, 0.40–0.59 “moderate”, 0.60–0.79 “strong”, 0.80–1.0 “very strong”.

## 3. Results

**Figure 1:**
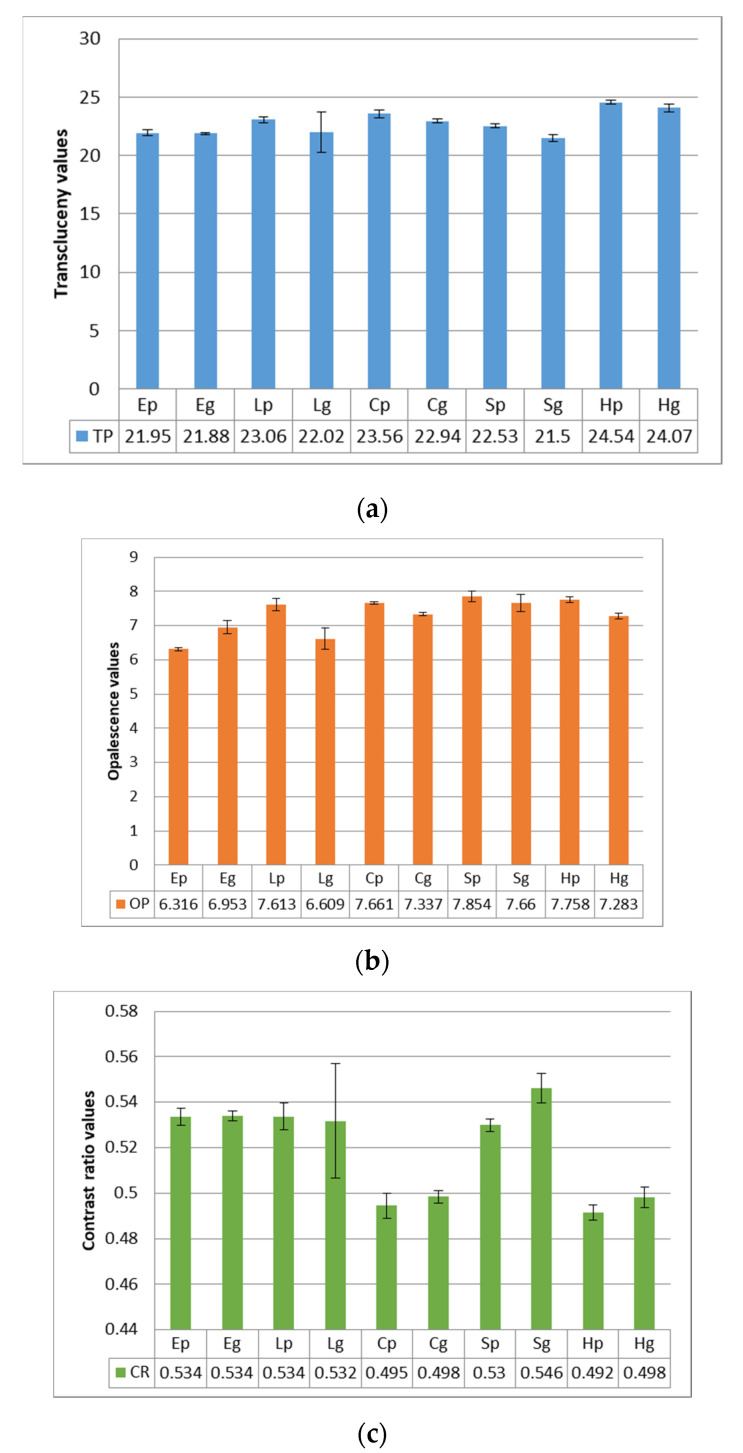
(**a**) Transclucency parameter average values of the tested samples with different try-in paste and substrate. (**b**) Opalescence parameter average values of the tested samples with different try-in paste and substrate. (**c**) Contrast ratio parameter average values of the tested samples with different try-in paste and substrate.

L*, a*, b* values were registered on white and black backgrounds for each group of polished [p] and glazed [g] samples, and TP, CR an OP values were calculated (Figure 1).

TP average values for all materials were in the range of 21.49–24.53. The decreasing order is: H > L > C > S > E, with significant differences (*p* < 0.005) between H and all other materials, between C and S, E. Polished samples were more translucent than glazed. OP average values were in the rage of 6.31–7.85. E and L exhibited similar values and the lowest opalescence, C and H likewise similar and higher, and s the highest. Polished samples are more opalescent than glazed, excepting for E. OP is moderate positive correlated to TP (r = 0.406). CR is negative and strong correlated to TP (r = −0.867).

**Figure 2:**
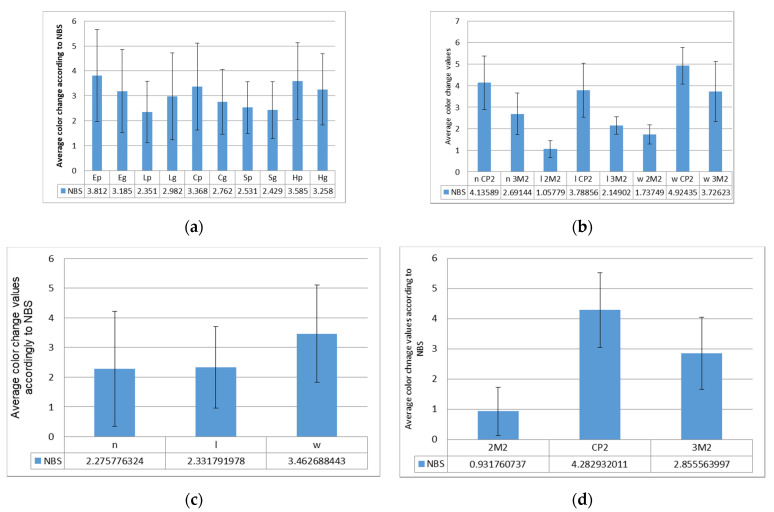
Average color changes and SD, related to (**a**) restoration material, (**b**) luting material/substrate, (**c**) luting material, and (**d**) substrate.

According to NBS units, perceivable color changes were calculated (Figure 2). Related to materials, average color changes decrease as following: E > H > C > L > S, with significant differences (*p* < 0.05) between E-S and H-L, H-S.

Marked color changes were calculated for all materials, in the following descending order: H > C > E > S > L. The percentage of slight changes decrease thus: L > S > E > H > C, and for perceivable changes: S > L > C > E > H.

## 4. Discussion

According to these results, the first null hypothesis “the masking ability of resin composites ceramic materials shows no significant differences” is rejected.

Referring to the try-in material, warm colors induce marked color changes of the restoration, significant higher than neutral or light colored try-in materials. A substrate color which differs from the restorative material color leads to marked color changes if it is darker or mainly with a higher chroma. The second null hypothesis refers to the luting agent, namely “the luting agent affects the color of the final restoration” is accepted.

The differences of the color changes determined by all studied substrates are significant.

According to the surface treatments, the polished surfaces proved to have higher translucency after optical evaluation, compared to the glazed samples that had higher opalescence. For the contrast ratio values, both polished and glazed samples proved to have the same behavior. Other study published their optical results showing that the glaze can influence the opalescence parameter, because increases the yellowness of the samples [47].

Taking into account both parameters, try-in pastes and substrates, marked color changes are related to CP2 substrates, irrespective of t try-in paste, followed by 3M2 substrate associated with w try-inpaste. 3M2 substrates associated with n or l try-in pastes lead to perceivable color changes. Extremely marked color change values of the materials, related to try-in pastes (n, l, w), and substrates (2M2, CP2, 3M2) were registered for Ep, Cp and Hp. The third null hypothesis is related to the substrate, “the color of the substrate influences its masking ability” and is accepted.

CAD/CAM achieved aesthetic monolithic restorations have been emphasized in order to reduce veneer chipping and to speed up the manufacturing process [48]. Therefore, a variety of aesthetic materials have been developed. The focus of the manufacturer is, in general, to include a relevant amount of crystal structures to ensure resistance, but in a moderate level to avoid a much opaque appearance. On the other hand high translucent materials generally are not able to mask the discolored substrates by their own potential and therefore this aspect must be taken into account in case of discolored substrates [49]

Reported mean TP values of 1 mm thick human enamel and human dentine were 18.7, and 16.4, respectively, for glass ceramics TP ranged from 14.9 to 19.6 and for zirconia from 5.5 to 13.5. In order to achieve aesthetics, the translucency of restorative materials has to be predictable for a given dental restoration. The translucency of composite materials and ceramics increase exponentially as the thickness decrease [50]. Adjustment of the translucency of aesthetic dental restorative materials has been investigated by the influence of filler size, amount on the difference between the transmitted and reflected colors [48,49,50]. The CR and TP values of aesthetic dental materials were compared in different studies. As a result, CR increased in inverse proportion to TP (correlation coefficient: r = −0.93). Mean CR values of enamel and dentine were negatively correlated with TP values (r = −0.93 to −0.78) [51,52]. Some studies suggested that the CR of dental ceramics is linearly related to the thickness. CR is a ratio of reflectance values [53,54].

The OP value of the enamel-dentin complex was reported to be 4.8, and that of enamel, 7.4 [55].

In respect to the optical properties of aesthetic materials, the present study assessed the specific effects of the association of three substrates with three shades of Try-in pastes, with different optical parameters, and different resin composites ceramic materials, considering a one body restoration, composed of substrate, luting agent, and restoration material. Related to TP, average value were registered to be in the range of 21.49–24.53, with higher statistical values for H and C. For the CR, which is negative and strong correlated to TP (r = −0.867), the same materials registered statistically lower values. As a result of these optical properties, the higher masking ability should be assigned to S, L, and E.

As limitations of this study, the involved try-in paste contains glycerin, pigments and silica (filler particles) that are added to simulate the shade properties of the analogues resin-based luting agents. However, there is a possibility that small differences in the final color might occur after the polymerization of the resin-based luting agent [56,57]. The shade agreement between the try-in pastes and the respective cements has been evaluated in different studies, some found no significant differences, but other found no color matching of resin cements and corresponding try-in pastes. Different ceramic systems can have different effects on try-in paste and resin cement agreement [58,59,60,61,62,63].

Regarding the interaction between restoration material and luting cement, in another study, the degree of veneer translucency was found to be more effective in masking the underlying discolored substrate than the luting agent and this might be due to the cement thickness, which is less than that of the restoration [64]. Based on another study it can be concluded that changing shades of luting agent could be an alternative to increasing the thickness of a high-translucency aesthetic material in order to enhance its masking ability [65].

This study found that only the warm colored try-in material could induce marked color changes of the restoration. The substrate color which differs from the restorative material color led to marked color changes if it is darker or mainly with a higher chroma. After the calculations of average color changes according to NBS, changes were registered to be from slight to extremely mark. Extremely marked and marked are associated with H, C, and E, and perceivable and slight for L and S. Considering their optical properties, the higher masking ability of L and S should be attributed to the lower translucency, the lower masking ability of H and C, to the higher translucency, but for E the relative masking ability is not correlated to the optical properties. Its different behavior should be attributed to the different microstructure, because E belongs to a polymer infiltrated ceramic, is not a nanoparticle infiltrated resin, like the other investigated materials.

In practice, the thickness of the restoration is restricted by the minimal amount of tooth preparation, the different resin cement shades are limited and might be selected to slightly modify the final color [66,67,68,69,70]. Discolored substrates are common and therefore practitioners have to consider which kind of aesthetic material can better mask them and recover the optical properties of natural teeth [71]. The limitations of this study were that it was an in vitro study, try-in pastes were used. Another limitation is that only one translucency of the resin composites ceramic materials was used.

## 5. Conclusions

Taking into account the limitations of this in vitro study, the following main conclusions were reached:

1. The underlying dental structure, luting agent, and restoration material have a significant impact on the aesthetic aspect of the restoration.
2. The masking ability was the best for Shofu HC and Lava Ultimate, followed by Cerasmart, and significant lower Hyramic and Vita Enamic.
3. Warm try-in pastes associated with non-discolored substrates can determine at most perceivable color changes, but associated with dark colored ones can lead to marked color changes.
4. For substrates with a darker color, warm try-in pastes lead to marked color changes, but neutral and light try-in pastes at most perceivable, if they are associated with resin composites ceramic materials which proved to have a better masking ability.

## Figures and Tables

**Table 1:** Composition and manufacturer specifications of tested materials [41,42].

Material	Type	Monomer	Filler
Vita Enamic HT [E] VITA Zahnfabrik, Bad Säckingen, Germany	Resin composites materials material	UDMA, TEGDMA	Feldspar ceramic enriched with aluminum oxide 86%
Lava Ultimate HT [L] 3M ESPE, Seefeld, Germany	CAD/CAM composite resin	Bis-GMA, UDMA, Bis-EMA, TEGDMA	SiO_2_, ZrO_2_, aggregated ZrO_2_/SiO_2_ cluster 80%
Cerasmart HT [C] GC Corporation, Tokyo, Japan	CAD/CAM composite resin	Bis-MEPP, UDMA, DMA	Silica, barium glass 71%
Shofu HC HT [S] Shofu, Kyoto, Japan	CAD/CAM composite resin	UDMA, TEGDMA	Silica, silicate, zirconium silicate 61%
Hyramic HT [H] Upcera, Liaoning, China	CAD/CAM composite resin	-	Inorganic Filler 55–85%

**Table 2:** Levels of color change, according to NBS.

NBS Units	Color Changes
0.0–0.5	extremely slight change
0.5–1.5	slight change
1.5–3.0	perceivable
3.0–6.0	marked change
6.0–12.0	extremely marked change
12.0 or more	change to another color

## Data Availability

Not applicable.

## References

[1] Seyidaliyeva A., Rues S. (2020). Color stability of polymer-infiltrated-ceramics compared with lithium disilicate ceramics and composite. J. Esthet. Restor. Dent..

[2] Coldea A., Swain M.V. (2013). Mechanical properties of polymer-infiltrated-ceramic-network materials. Dent. Mater..

[3] He L.H., Swain M. (2011). A novel polymer infiltrated ceramic dental material. Dent. Mater..

[4] Ashtiani A.H., Azizian M. (2019). Comparison the degree of enamel wear behavior opposed to polymer-infiltrated ceramic and feldspathic porcelain. Dent. Res. J..

[5] Yin R., Kim Y.K. (2019). Comparative evaluation of the mechanical properties of CAD/CAM dental blocks. Odontology.

[6] Choi J.W., Song E.J. (2017). In vitro investigation of wear of CAD/CAM polymeric materials against primary teeth. Materials.

[7] Xu Z., Yu P. (2017). A comparative study on the wear behavior of a polymer infiltrated ceramic network (PICN) material and tooth enamel. Dent. Mater..

[8] Skorulska A., Piszko P. (2021). Review on Polymer, Ceramic and Composite Materials for CAD/CAM Indirect Restorations in Dentistry-Application, Mechanical Characteristics and Comparison. Materials.

[9] Manziuc M.M., Gasparik C. (2021). Color and masking properties of translucent monolithic zirconia before and after glazing. J. Prosthodont. Res..

[10] Araujo E., Perdigao J., Perdigao J. (2016). Restorative options for discoloured teeth. Tooth Whitening.

[11] Zhi L., Bortolotto T. (2016). Comparative in vitro wear resistance of CAD/CAM composite resin and ceramic materials. J. Prosthet. Dent..

[12] Bacchi A., Boccardi S. (2019). Substrate masking ability of bilayer and monolithic ceramics used for complete crowns and the effect of association with an opaque resin-based luting agent. J. Prosthodont. Res..

[13] López-Suárez C., Castillo-Oyagüe R. (2018). Fracture load of metal-ceramic, monolithic, and bi-layered zircônia-based posterior fixed dental prosthesis after thermo-mechanical cycling. J. Dent..

[14] Belli R., Wendler M., de Ligny D., Cicconi M.R., Petschelt A., Peterlik H., Lohbauer U. (2017). Chairside CAD/CAM materials: Part 1: Measurement of elastic constants and microstructural characterization. Dent. Mater..

[15] Begum Z., Chheda P. (2014). Effect of Ceramic Thickness and Luting Agent Shade on the Color Masking Ability of Laminate Veneers. J. Indian Prosthodont. Soc..

[16] Vichi A., Ferrari M. (2000). Influence of ceramic and cement thickness on the masking of various types of opaque posts. J. Prosthet. Dent..

[17] Tabatabaian F., Javadi Sharif M. (2017). The color masking ability of a zirconia ceramic on the substrates with different values. J. Dent. Res. Dent. Clin. Dent. Prospect..

[18] Rafael C.F., Del Pina Luna M., Munõz M.T., Garbelotto D.A., Gustavo L., Liebermann A., Özcan M., Maziero Volpato C.Â. (2017). Optical Factors: Affecting Anterior Esthetics in All-Ceramic Restorations: Two Case Reports. J. Cosmet. Dent..

[19] Turgut S., Bagis B., Ayaz E.A. (2014). Achieving the desired colour in discoloured teeth, using leucite-based cad-cam laminate Systems. J. Dent..

[20] Daneshpooy M., Pournaghi Azar F. (2019). Color agreement between try-inpaste and resin cement: Effect of thickness and regions of ultra-translucent multilayered zirconia veneers. J. Dent. Res. Dent. Clin. Dent. Prospect..

[21] Lee Y.K. (2016). Opalescence of human teeth and dental esthetic restorative materials. Dent. Mater. J..

[22] Lee Y.K., Powers J.M. (2006). Influence of opalescence and fluorescence properties on the light transmittance of resin matrix ceramic materials as a function of wavelength. Am. J. Dent..

[23] Lee Y.K., Lu H. (2006). Influence of fluorescent and opalescent properties of resin matrix ceramic materialss on the masking effect. J. Biomed. Mater. Res. B Appl. Biomater..

[24] Tabatabaian F., Dalirani S. (2019). Effect of Thickness of Zirconia Ceramic on Its Masking Ability: An In Vitro Study. J. Prosthodont..

[25] Vichi A., Sedda M. (2016). Comparison of contrast ratio, translucency parameter, and flexural strength of traditional and “Augmented translucency” zirconia for CEREC CAD/CAM system. J. Esthet. Restor. Dent..

[26] Suputtamongkol K., Tulapornchai C. (2013). Effect of the shades of background substructures on the overall color of zirconia-based all-ceramic crowns. J. Adv. Prosthodont..

[27] Choi Y.J., Razzoog M.E. (2013). Masking ability of zirconia with and without veneering porcelain. J. Prosthodont..

[28] Tabatabaian F., Masoomi F. (2016). Effect of three different core materials on masking ability of a zirconia ceramic. J. Dent..

[29] Oh S.H., Kim S.G. (2015). Effect of abutment shade, ceramic thickness, and coping type on the final shade of zirconia all-ceramic restorations: In vitro study of color masking ability. J. Adv. Prosthodont..

[30] Sinmazisik G., Demirbas B. (2014). Influence of dentin and core porcelain thickness on the color of fully sintered zirconia ceramic restorations. J. Prosthet. Dent..

[31] Son H.J., Kim W.C. (2010). Influence of dentin porcelain thickness on layered all-ceramic restoration color. J. Dent..

[32] Selz C.F., Bogler J. (2015). Veneered anatomically designed zirconia FDPs resulting from digital intraoral scans: Preliminary results of a prospective clinical study. J. Dent..

[33] Pop-Ciutrila I.S., Dudea D. (2016). Shade correspondence, color, and translucency differences between human dentine and a CAD/CAM hybrid ceramic system. J. Esthet. Restor. Dent..

[34] Salameh Z., Tehini G. (2014). Influence of ceramic color and translucency on shade match of CAD/CAM porcelain veneers. Int. J. Esthet. Dent..

[35] Kurklu D., Azer S.S. (2013). Porcelain thickness and cement shade effects on the colour and translucency of porcelain veneering materials. J. Dent..

[36] Skyllouriotis A.L., Yamamoto H.L. (2017). Masking properties of ceramics for veneer restorations. J. Prosthet. Dent..

[37] Kurtulmus-Yilmaz S., Cengiz E. (2019). The effect of surface treatments on the mechanical and optical behaviors of CAD/CAM restorative materials. J. Prosthodont..

[38] Awad D., Stawarczyk B. (2015). Translucency of esthetic dental restorative CAD/CAM materials and composite resins with respect to thickness and surface roughness. J. Prosthet. Dent..

[39] Su Y., Xin M., Chen X. (2021). Effect of CAD-CAM ceramic materials on the color match of veneer restorations. J. Prosthet. Dent..

[40] Horvath S.D. (2016). Key Parameters of Hybrid Materials for CAD/CAM-Based Restorative Dentistry. Compend. Contin. Educ. Dent..

[41] Alamoush R.A., Silikas N. (2018). Effect of the Composition of CAD/CAM Composite Blocks on Mechanical Properties. Biomed. Res. Int..

[42] Johnston W.M. (2014). Review of translucency determinations and applications to dental materials. J. Esthet. Res. Dent..

[43] Porojan L., Vasiliu R.-D. (2020). Surface Characterization and Optical Properties of Reinforced Dental Glass-Ceramics Related to Artificial Aging. Molecules.

[44] Shirani M., Savabi O. (2021). Comparison of translucency and opalescence among different dental monolithic ceramics. J. Prosthet. Dent..

[45] Agarwal M., Wible E. (2018). Long-term effects of seven cleaning methods on light transmittance, surface roughness, and flexural modulus of polyurethane retainer material. Angle Orthod..

[46] Fang D., Zhang N., Chen H. (2013). Dynamic stress relaxation of orthodontic thermoplastic materials in a simulated oral environment. Dent. Mater. J..

[47] Nguyen-Tri P., Prud’homme R.E. (2019). Nanoscale analysis of the photodegradation of Polyester fibers by AFM-IR. J. Photochem. Photobiol. A Chem..

[48] Biron M. (2018). Thermoplastics and Thermoplastic Composites. Detailed Accounts of Thermoplastic Resins.

[49] Porojan L., Vasiliu R.-D. (2020). Surface Quality Evaluation of Removable Thermoplastic Dental Appliances Related to Staining Beverages and Cleaning Agents. Polymers.

[50] Wang F., Takahashi H., Iwasaki N. (2013). Translucency of dental ceramics with different thicknesses. J. Prosthet. Dent..

[51] Lee Y.-K. (2015). Translucency of human teeth and dental restorative materials and its clinical relevance. J. Biomed. Opt..

[52] Akar G.C. (2014). Effects of surface-finishing protocols on the roughness, color change, and translucency of different ceramic systems. J. Prosthet. Dent..

[53] Monaco C., Arena A. (2014). Effect of prophylactic polishing pastes on roughness and translucency of lithium disilicate ceramic. Int. J. Periodontics Restor. Dent..

[54] Lee S.H., Lee Y.K. (2004). Influence of thermocycling on the optical properties of laboratory resin matrix ceramic materialss and an all-ceramic material. J. Mater. Sci. Mater. Med..

[55] Yu B., Ahn J.S. (2009). Measurement of translucency of tooth enamel and dentin. Acta Odontol. Scand..

[56] Antonson S.A., Anusavice K.J. (2001). Contrast ratio of veneering and core ceramics as a function of thickness. Int. J. Prosthodont..

[57] Vasiliu R.-D., Porojan S.D. (2020). Effect of Thermocycling, Surface Treatments and Microstructure on the Optical Properties and Roughness of CAD-CAM and Heat-Pressed Glass Ceramics. Materials.

[58] Ardu S., Feilzer A.J. (2008). Quantitative clinical evaluation of esthetic properties of incisors. Dent. Mater..

[59] ALGhazali N., Laukner J. (2010). An investigation into the effect of trytry-inpastes, uncured and cured resin cements on the overall color of ceramic veneer restorations: An in vitro study. J. Dent..

[60] Vaz E.C., Vaz M.M. (2019). Resin Cement: Correspondence with Try-inPaste and Influence on the Immediate Final Color of Veneers. J. Prosthodont..

[61] Xing W., Jiang T. (2010). Evaluation of the esthetic effect of resin cements and try-in pastes on ceromer veneers. J. Dent..

[62] Vaz E., Vaz M. (2016). Try-inPastes Versus Resin Cements: A Color Comparison. Compend. Contin. Educ. Dent..

[63] Rigoni P., Amaral F.L.B.D. (2012). Color agreement between nanofluorapatite ceramic discs associated with try-inpastes and with resin cements. Braz. Oral Res..

[64] Xu B., Chen X. (2014). Agreement of Try-In Pastes and the Corresponding Luting Composites on the Final Color of Ceramic Veneers. J. Prosthodont..

[65] Mourouzis P., Koulaouzidou E. (2018). Color match of luting composites and try-inpastes: The impact on the final color of CAD/CAM lithium disilicate restorations. Int. J. Esthet. Dent..

[66] Kandil B.S.M., Hamdy A.M. (2019). Effect of ceramic translucency and luting cement shade on the color masking ability of laminate veneers. Dent. Res. J..

[67] Dai S., Chen C. (2019). Choice of resin cement shades for a high-translucency zirconia product to mask dark, discolored or metal substrates. J. Adv. Prosthodont..

[68] Xing W., Chen X. (2017). The effect of ceramic thickness and resin cement shades on the color matching of ceramic veneers in discolored teeth. Odontology.

[69] Azer S.S., Rosenstiel S.F. (2011). Effect of substrate shades on the color of ceramic laminate veneers. J. Prosthet. Dent..

[70] Della Bona A., Pecho O.E. (2015). Colour parameters and shade correspondence of CAD-CAM ceramic systems. J. Dent..

[71] Kim H.K., Kim S.H. (2013). Effect of polishing and glazing on the color and spectral distribution of monolithic zirconia. J. Adv. Prosthodont..

